# Common Data Elements for Acute Coronary Syndrome: Analysis Based on the Unified Medical Language System

**DOI:** 10.2196/14107

**Published:** 2019-08-23

**Authors:** Markus Kentgen, Julian Varghese, Alexander Samol, Johannes Waltenberger, Martin Dugas

**Affiliations:** 1 Institute of Medical Informatics University of Münster Münster Germany; 2 Medical Faculty University Hospital of Münster Münster Germany

**Keywords:** common data elements, acute coronary syndrome, documentation, standardization

## Abstract

**Background:**

Standardization in clinical documentation can increase efficiency and can save time and resources.

**Objective:**

The objectives of this work are to compare documentation forms for acute coronary syndrome (ACS), check for standardization, and generate a list of the most common data elements using semantic form annotation with the Unified Medical Language System (UMLS).

**Methods:**

Forms from registries, studies, risk scores, quality assurance, official guidelines, and routine documentation from four hospitals in Germany were semantically annotated using UMLS. This allowed for automatic comparison of concept frequencies and the generation of a list of the most common concepts.

**Results:**

A total of 3710 forms items from 86 sources were semantically annotated using 842 unique UMLS concepts. Half of all medical concept occurrences were covered by 60 unique concepts, which suggests the existence of a core dataset of relevant concepts. Overlap percentages between forms were relatively low, hinting at inconsistent documentation structures and lack of standardization.

**Conclusions:**

This analysis shows a lack of standardized and semantically enriched documentation for patients with ACS. Efforts made by official institutions like the European Society for Cardiology have not yet been fully implemented. Utilizing a standardized and annotated core dataset of the most important data concepts could make export and automatic reuse of data easier. The generated list of common data elements is an exemplary implementation suggestion of the concepts to use in a standardized approach.

## Introduction

Acute coronary syndrome (ACS), with its three subforms—ST elevated myocardial infarction (STEMI), non-ST elevated myocardial infarction (NSTEMI), and unstable angina pectoris (UAP)—is among the leading causes of mortality around the world [1,2]. Studies estimate that more than 3 million people worldwide each year get diagnosed with STEMI and more than 4 million with NSTEMI [3].

Documentation is an important and required part of patient care. For patients with ACS, data collection often starts in the emergency department and can continue beyond the discharge date, when documentation for quality assurance or research purposes is needed. Several studies have found that documentation takes a significant part of a physician’s time, with findings ranging from a quarter to half of available daily work time [4-7]. At the same time, documentation has been found to be lacking important information [8], with potentially dangerous effects for patients [9]. Parallel and redundant documentation for uses other than routine patient care, such as quality measurements or research and patient registries, make this process even more time-consuming and can result in documentation inconsistencies or errors. Over the past several years, spending for data management in research studies has increased. Implementing standardized documentation approaches also has financial benefits [10].

One way of ensuring standardized documentation is through the use of common data elements (CDEs), which the National Institutes of Health defines as “A data element that is common to multiple data sets across different studies” [11]. Use of CDEs in a semantically annotated and machine-readable format facilitates comparison and aggregation of data across studies, independent from language; ensures data consistency; and simplifies sharing of research results [12-15].

In a context of clinical decision support systems (CDSS), which have the potential to improve quality of care [16], clear definition of necessary concepts is essential. Lack of data availability has been shown to be one of the main obstacles in creating and using CDSS [17]. Lack of standardization forces each implementation to develop its own data model [18].

Official guidelines for STEMI and NSTEMI patients were published by the European Society for Cardiology (ESC) [19,20] and the American Heart Association/American College of Cardiology (AHA/ACC) [21,22]. These guidelines mention several data concepts required for patient care, but do not explicitly define them. Both the ESC [23] and the American Heart Association (AHA), together with the American College of Cardiology Foundation (ACCF) [24,25] also have made official recommendations for key data elements in documentation for patients with ACS, which lack semantic annotation.

The aim of our work is to conduct a semiautomated approach, in which forms from different documentation contexts (ie, routine patient care and research and quality assurance) were semantically compared to build a set of CDEs based on concept frequency and allowed analysis of similarities and standardization within forms.

## Methods

### Definition of Documentation Contexts and Form Collection

Based on a workflow already successfully used for acute myeloid leukemia [26], a set of five documentation contexts in which information is collected for ACS patients was defined: routine documentation, research (ie, registries and clinical studies), quality measurements, official recommendations, and clinical risk scores.

Forms from each context were collected between March and December 2015. Relevant studies and registries have been identified by a PubMed and Google Scholar search using the following keywords: “acute coronary syndrome,” “myocardial infarction,” “angina,” “angina pectoris,” “chest pain,” “ST elevation myocardial infarction,” “non-ST elevation myocardial infarction + registry,” “cohort,” “data set,” “documentation,” “quality measures,” and “guideline.”

Forms used in routine patient care were collected from three university hospitals—Dresden, Magdeburg, and Münster—and one nonuniversity hospital—Bremen—in Germany. All forms containing information, which later gets included in the patient’s data record, were included. This includes, but is not limited to, all documentation on patient history, diagnostics (eg, electrocardiogram [ECG] and lab results), examination results, therapeutic procedures (eg, percutaneous coronary intervention), and medication.

To gain access to the case report forms (CRFs) of two selected studies conducted by pharmaceutical companies, an inquiry to receive the forms was made to, and granted by, the European Medicines Agency. To get a broader summary of the concepts relevant for study documentation, we also incorporated the inclusion and exclusion criteria of all studies listed on ClinicalTrials.gov that were completed after January 1, 2010, were tagged with “acute coronary syndrome,” and were shown to have results. Principal investigators of selected large registries with relevance for ACS have been contacted and asked for their permission for us to use the CRFs for analysis.

A total of 10 risk scores, or scores for outcome prediction, as well as the officially recommended key datasets from the American Heart Association/American College of Cardiology Foundation (AHA/ACCF) [24] and the ESC [23] were included as well.

### Semantic Form Annotation

All forms were manually transformed into Operational Data Model (ODM) files. Each data item from those forms was assigned an item name, a data type, and a suggested value set, whenever applicable; each data item was then semantically annotated with Unified Medical Language System (UMLS) codes by a medical expert.

Established coding principles [27] were used to assign medical concepts and corresponding UMLS codes to each form item. The medical concept is a semantic identifier to encode the medical information that is required by the item (eg, the item “Creatinine >4 mg/dL” is encoded by the concept “Creatinine measurement,” UMLS code: C0201976). Whenever possible, each form item was assigned a single existing (ie, precoordinated) UMLS code. If no precoordinated code existed, we attempted to describe each item with no more than two different UMLS codes. If this failed, no code was assigned.

For example, for a form item “Patient date of birth,” the precoordinated code C0001779 (“Date of birth”) could be assigned. A precoordinated code for “Location of bleeding” does not exist, so the concept was postcoordinated as “C0019080 C0450429” (“Hemorrhage” and “Location”). The UMLS metathesaurus [28] was used to find the appropriate codes; the use of the ODM editor [29] allowed for reuse of UMLS codes already assigned in other forms by suggesting appropriate codes based on similarity of item questions and item names.

Nondistinct data items (eg, “Other medication” and “Other comments”) and items containing study internals or administrative data and, therefore, no relevant medical concept (eg, “Technician id”) have been discarded in the process.

### Creation of Common Data Element List

All encoded forms were then automatically compared and analyzed using CDEGenerator [30]. The Web application generated a list of all UMLS codes, their absolute and relative frequencies in different documentation contexts, and an overview of original questions and form occurrence.

**Figure 1:**
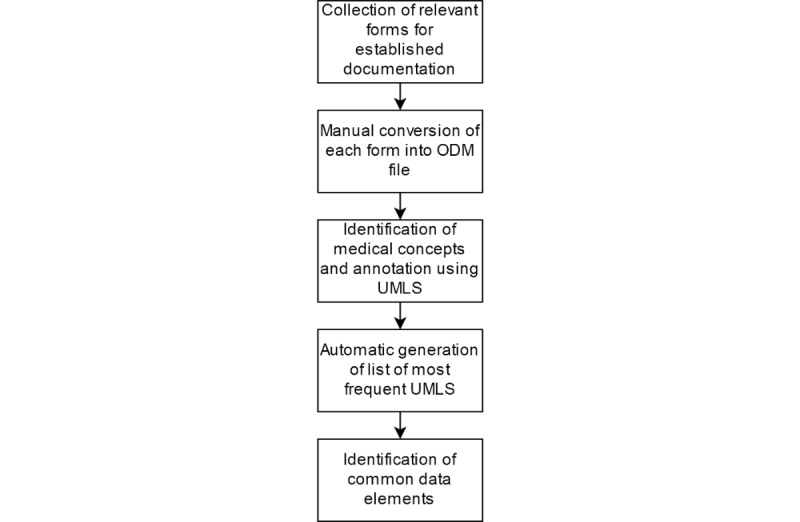
Form collection, semantic enrichment, and semantic analyses. ODM: Operational Data Model; UMLS: Unified Medical Language System.

In a manual code cleaning step, all assigned codes were then reviewed manually to ensure that different codes were not used for identical medical concepts. The resulting list was then also reviewed by a second medical expert to revise incorrect code assignments. About 1% of codes were changed manually. For easier readability, concepts were sorted into categories—patient data, timepoints, patient history, laboratory, medication, procedures, examinations, diagnosis, ECG, and outcome—and double-checked by a cardiologist for clinical relevance and missing concepts. For the top list, we also checked that every concept occurs in at least two documentation contexts. Concepts that appeared only once in all analyzed forms were removed from the final list. Figure 1 illustrates the individual steps of the process.

A more detailed analysis of differences between the documentation contexts (eg, differences between routine and research documentation) was done by merging all forms of one context into a single ODM file. By entering the merged files into CDEGenerator, pairwise comparison between two contexts was possible. The resulting output shows unique and shared concepts between two contexts and allows for calculation of overlap percentages.

## Results

### Overview

A total of 15 research groups have been asked to provide their CRFs. Out of the 15 groups, 3 (20%) of them sent the corresponding forms to us; the others either did not reply or refused our request. All contacted research groups and their responses are listed in Multimedia Appendix 1. Four other registry forms were publicly available and were included.

A total of 86 forms have been included in the analysis. Table 1 shows the distribution of forms along documentation contexts. The full list of all forms can be found in Multimedia Appendix 2. In these forms, 3710 medical concepts have been identified. For 3637 out of the 3710 concepts (98.03%), a suitable UMLS annotation could be found. A total of 842 unique UMLS concepts were used in the annotation process; 52 of them (6.2%) were postcoordinated.

**Table 1:** Overview of analyzed sources.

Documentation context	Number of sources	Sources
Routine documentation	3	University hospitals
	1	Nonuniversity hospital
Research	7	Registries
	2	Studies, all case report forms
	34	Studies, eligibility criteria
Quality measurements	6	N/A^a^
Recommendations from official associations	2	N/A
Risk and outcome scores	10	N/A

^a^N/A: not applicable.

**Figure 2:**
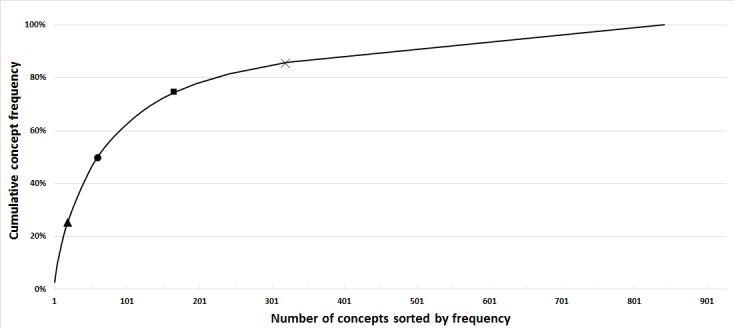
Cumulative code frequencies. Starting with the most common concept, absolute frequencies of all concepts were cumulatively added. The 60 most common concepts cover 50% of all concept occurrences (circle). The first 18 concepts cover 25% (triangle) and the first 167 concepts cover 75% of all occurrences (square). After 321 concepts, each concept occurs only once and the graph increases linearly (cross).

Cumulative frequencies were calculated to assess the heterogeneity of concepts. Figure 2 displays the results, showing that the 60 most frequent unique concept codes out of 842 (7.1%) are sufficient to cover 50% of all concept occurrences.

For about 2% of all form items, neither a suitable precoordinated concept could be found, nor was it possible to create a postcoordinated concept. In most cases, this is due to high complexity, which made it difficult to apply unambiguous postcoordination. For example, a form question like “Were any stents placed to the target vessel of the index event?” proved to be too complex for postcoordination and could not be reduced to a single existing UMLS code without altering its meaning.

No precoordinated UMLS codes could be found for “Door-to-balloon time,” “Time of first medical contact,” “Main trunk stenosis,” and “Dose area product,” although they are quality markers for ACS in German quality assurance.

### Common Data Elements

The most frequently used concept is “Percutaneous coronary intervention (PCI),” with an absolute frequency of 98, followed by “Stroke” with a frequency of 77 and “Date of birth” with a frequency of 73. Absolute frequencies can be higher than the total number of forms because concepts can appear more than once per form, for example, when asked in different subforms at different points in time (eg, at follow-up).

Table 2 shows the 10 most common concepts, their absolute and relative frequencies, subconcepts, and the suggested UMLS codes. The complete list of concepts sorted by frequency can be found in Multimedia Appendix 3.

**Figure 3:**
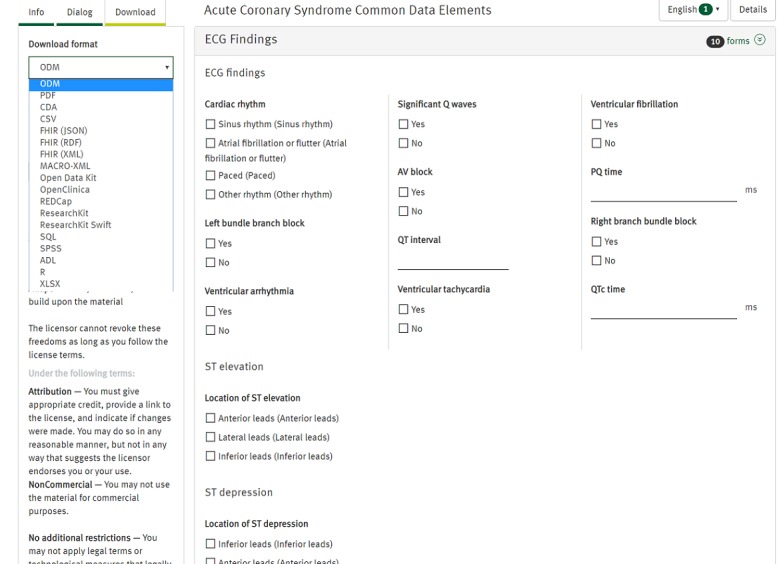
Extract of resulting common data elements (CDEs) for electrocardiogram (ECG) findings, which is exportable in various data formats.

The revised list of CDEs can be found in Multimedia Appendix 4 and has open-access availability on the Medical Data Models portal [31] with a number of conversions available, such as REDCap models or Health Level Seven (HL7) Fast Healthcare Interoperability Resources (FHIR) questionnaire [32]. Figure 3 shows an exemplary screenshot of the ECG section of the CDEs together with the export function. This enables easy reuse of the resulting data concepts within other medical documentation systems and export into various other standard formats.

**Table 2:** Top 10 most frequent concepts by absolute and relative frequency with subconcepts, a suggested semantic Unified Medical Language System (UMLS) annotation, and occurrence across documentation contexts.

Concept name	Subconcepts	Suggested UMLS code	Afreq^a^, n	Rfreq^b^, %	Documentation context				
					Routine	Research	QA^c^	ORec^d^	Scores
PCI^e^	History of PCI or revascularization Total numbers of PCI procedures Date of PCI Indication for PCI Contraindication for PCI	C1532338	98	2.6	X	X	X	X	
Stroke or TIA^f^	History of stroke or TIA Date of stroke or TIA Type of stroke: hemorrhagic or ischemic	C0038454	77	2.0	X	X	X	X	
Date of birth	N/A^g^	C0001779	73	2.0	X	X	X	X	X
Hemorrhage other than stroke	Hemorrhage location Hemorrhage intensity: major or minor Date of hemorrhage History of hemorrhage Treatment or reoperation due to bleeding	C0019080	69	1.9		X	X	X	
CABG^h^	History of CABG Date of most recent CABG	C0010055	53	1.4	X	X	X	X	
Angina pectoris	CCS^i^ classification History of angina pectoris	C0002962	48	1.3	X	X	X	X	
Blood pressure	Systolic blood pressure Diastolic blood pressure	C0871470	47	1.3	X	X		X	X
Death	Patient died Date of death Cause of death	C1306577	47	1.3		X	X	X	
Coronary angiography	Date of coronary angiography Indication for coronary angiography Contraindication for coronary angiography	C0085532	46	1.2	X	X	X	X	
MI^j^	History of MI Date of most recent MI Acute MI Isolated posterior MI Recurrent MI Posterior infarction	C0027051	44	1.2	X	X	X	X	

^a^Afreq: absolute frequency.

^b^Rfreq: relative frequency.

^c^QA: quality assurance.

^d^ORec: official recommendations.

^e^PCI: percutaneous coronary intervention.

^f^TIA: transient ischemic attack.

^g^N/A: not applicable.

^h^CABG: coronary artery bypass graft.

^i^CCS: Canadian Cardiovascular Society.

^j^MI: myocardial infarction.

### Comparison of Documentation Contexts

Comparison of concepts in routine documentation (323 unique concepts), registries (340 unique concepts), pharmacological studies (257 concepts), and eligibility criteria (166 concepts) shows overlap percentages ranging from 17% to 33%. Comparison of the suggested key data elements from the Cardiology Audit and Registration Data Standards (CARDS) by the ESC [23] (82 concepts) and the AHA/ACCF [24] (153 concepts) shows 51 matching concepts, which means that 62% (51/82) of the CARDS concepts exist in the AHA/ACCF key dataset and 33.3% (51/153) of the concepts of the AHA/ACCF dataset can be found in CARDS. Table 3 shows the complete results of the overlap analysis.

Table 4 shows the results of a comparison of concepts between the four analyzed hospitals. With 111, 110, 114, and 101 unique concepts, all hospitals collect about the same amount of data. Between 37 and 53 of them are matching in pairwise comparison. A total of 32 concepts do appear in documentation of all four hospitals. Those are primarily basic patient concepts, such as “Date of birth,” “Patient name,” “Diagnosis,” or “Date of admission,” as well as laboratory and examination results (eg, “Creatinine,” “Blood pressure,” or “Heart rate”).

Both data standards (184 unique concepts) have 69 concepts in common with routine documentation of all hospitals (323 unique concepts), which equates to overlap percentages of 37.5% and 21.4%, respectively.

Low overlap mainly results from frequent use of free-text fields in routine patient documentation. This also is the main difference between the documentation contexts. Research documentation, in general, uses more specific concepts (eg, asks directly for dosage, time, and contraindications for a drug), whereas routine documentation consists mainly of broader concepts (eg, “Medication list” instead of directly mentioning specific drugs).

**Table 3:** Overlap percentages between documentation contexts^a^.

Set 1, number of concepts	Set 2, number of concepts	Number of mutual concepts	Relative overlap in Set 1, %	Relative overlap in Set 2, %
ESC^b^ and CARDS^c^, 82	AHA/ACCF^d^, 153	51	62	33
Registries, 340	Risk scores, 46	34	10	74
Registries, 340	QA^e^, 64	48	14	75
Registries, 340	Pharmacological studies, 257	102	30	40
Registries, 340	Eligibility criteria, 166	79	23	48
Registries, 340	Routine documentation, 323	96	28	30
Registries, 340	Standards, 184	135	40	73
QA, 64	Risk scores, 46	10	16	22
QA, 64	Pharmacological studies, 257	28	44	11
QA, 64	Eligibility criteria, 166	26	41	16
QA, 64	Routine documentation, 323	27	42	8
QA, 64	Standards, 184	37	58	20
Pharmacological studies, 257	Risk scores, 46	27	11	59
Pharmacological studies, 257	Eligibility criteria, 166	69	27	42
Pharmacological studies, 257	Routine documentation, 323	62	24	19
Pharmacological studies, 257	Standards, 184	85	33	46
Routine documentation, 323	Risk scores, 46	25	8	54
Routine documentation, 323	Eligibility criteria, 166	55	17	33
Routine documentation, 323	Standards, 184	69	21	38
Eligibility criteria, 166	Standards, 184	64	39	35
Eligibility criteria, 166	Risk scores, 46	23	14	50
Risk scores, 46	Standards, 184	32	70	17

^a^Each documentation context was compared with all other contexts. The table lists the total number of concepts for each context as well as absolute and relative overlaps. For example, the second row compares the dataset of all registries with the dataset of all risk scores. A total of 340 unique concepts appear in all registries and 46 unique concepts appear in all risk scores. A total of 34 unique concepts appear in registries as well as in risk scores, which equates to a relative overlap of 10.0% (34/340) for registries and 74% (34/46) for scores.

^b^ESC: European Society for Cardiology.

^c^CARDS: Cardiology Audit and Registration Data Standards.

^d^AHA/ACCF: American Heart Association/American College of Cardiology Foundation.

^e^QA: quality assurance.

**Table 4:** Routine documentation compared between all four analyzed hospitals^a^.

Set 1, number of concepts	Set 2, number of concepts	Number of mutual concepts	Relative overlap in Set 1, %	Relative overlap in Set 2, %
Bremen, 111	Dresden, 110	47	42.3	42.7
Bremen, 111	Magdeburg, 114	43	38.7	37.7
Bremen, 111	Münster, 101	37	33.3	36.6
Dresden, 110	Magdeburg, 114	53	48.1	46.5
Dresden, 110	Münster, 101	46	41.8	45.5
Magdeburg, 114	Münster, 101	43	37.7	42.6

^a^The table lists the total number of unique concepts for each hospital and overlap percentages between the hospitals. For example, the first row compares the dataset of the routine documentation from the hospital in Bremen (111 concepts) with the dataset from the hospital in Dresden (110 concepts). A total of 47 unique concepts appear in both, which equates to a relative overlap of 42.3% (47/111) for Bremen and 42.7% (47/110) for Dresden.

## Discussion

### Principal Findings

Sizes of the different contexts differed noticeably. Scores do, of course, only consist of a small number of concepts (ie, 5-14), whereas big pharmacological studies tend to consist of several pages of documentation (ie, 310 and 514 concepts). The need to complete documentation of this size for each patient may very well explain increases of time and expenses for pharmacological studies [33]. Size of the routine documentation was found to be consistent between hospitals.

Overlap percentages between documentation contexts in general were found to be relatively low. This is partly to be expected since there are different areas of interest in different contexts of documentation. Pharmacological studies, for example, may need different information than patient registries. In addition, direct comparison of forms is complicated by differences in level of abstraction in questioning. For example, troponin blood levels are sometimes further differentiated between type I and T and sometimes are only referred to as “Troponin”; sometimes a form only asks for “elevated cardiac markers.” Although all share a common meaning, automated comparison or conversion is limited.

Although the benefits of annotated, machine-readable documentation have previously been established, none of the analyzed forms utilize semantically enriched data. All concepts were either identified by an internal ID code or the concept title, usually in English. Registries and studies already have a structure that in most cases would allow for an easy implementation of semantic annotation. Routine documentation, on the other hand, is more heterogeneous between different hospitals and consists of many free-text fields, which makes it more difficult to add semantic annotation.

CDSS rely on availability of machine-interpretable and -annotated patient data. Providing data, especially those required by the official guidelines, in such a manner could lead to improved quality of care. However, 50% of used concepts across all analyzed forms can be described by 60 UMLS codes. This indicates that a relatively small core dataset exists across all documentation contexts for ACS. Establishing documentation that includes these concepts in a semantically annotated format could make automatic export and reuse of data easier and would reduce redundancy and possibility for errors during transfer [34].

### Data Standards and Guidelines

Official patient care guidelines make use of several concepts as the basis for diagnosis or treatment decisions and risk stratification for patients with ACS. A complete list of all mentioned concepts or an explicit definition of all used data elements is not provided by the guidelines.

The key data elements suggested by the AHA/ACCF consist of more than 300 form questions. This by far exceeds the size of each registry or routine documentation analyzed. Overlap between official standards and routine documentation is also relatively small (ie, 38%). The lack of implementation indicates that a list of 300 concepts may be too extensive for physicians in a routine patient care environment to fill out. Some concepts that do not appear in any other form, such as “Pre-arrival first medical contact date/time,” may also lack importance, may be not available, or may be too hard to gather in routine patient care environments.

### Routine Documentation

Although all analyzed hospitals use about the same number of concepts (ie, 118, 134, 145, and 154) in their documentation process, less than half of these concepts are used in all hospitals. One reason for the small overlap may be the frequent use of free-text fields in routine documentation. Semantic annotation of data in free-text fields is difficult and, therefore, reuse of this data is limited. It is questionable if only 46% of the concepts in the officially recommended key data elements are used in routine care or if more of the recommended data could be found in nonmachine-readable free-text fields.

A total of 33% of concepts in routine documentation are part of the eligibility criteria analyzed, which is comparable to the findings of other studies [35]. This shows how little information is available for automatic patient recruitment.

### Suggested Implementation

Implementation of a semantically annotated set of CDEs into all contexts of documentation would allow automatic export of patient data for research and quality assurance, easy comparison of research data, and meta-analysis. Official suggestions for key data elements have been made [23]. They are, however, very comprehensive and, to date, only partly implemented. Although all suggested concepts may be important, an approach focused on a smaller set of concepts may be more suitable, especially for routine care providers. A balance needs to be found between the use of free text, which is difficult to reuse, and semantically annotated items, which are less flexible and more time-consuming initially but more valuable for secondary use. The frequency analysis performed, together with the recommendations by the official associations, could help in finding a suitable set.

### Limitations

UMLS is not a classification, meaning that there can be several synonymous codes representing the same medical concept. Although concept coding was done with great care and results have been checked by a second medical expert, annotation errors due to the ambiguity in the UMLS nomenclature cannot be completely ruled out.

During form collection, we attempted to get a representative sample of the documentation landscape for ACS. Since some requests to get forms for analysis were unsuccessful and analysis of routine documentation was only done with forms from hospitals in Germany, a selection bias could exist. Also, form collection was not done in a systematic way but, rather, was based on form availability; therefore, form number as well as item number per documentation context are not equal in size, which in theory allows for a selection bias or could have resulted in a different CDE list.

### Conclusions

The analysis shows a lack of standardization and semantic annotation in documentation of patients with ACS. Routine documentation, especially, frequently uses free text and makes easy export and reuse of data difficult. The results also suggest the existence of a relatively small core dataset that appears across many of the analyzed forms. Implementing semantically annotated CDEs based on this core dataset may reduce the time required for documentation and save money in the long run, although the clinical application remains to be tested.

## Appendix

Multimedia Appendix 1 List of documentation sources.

Multimedia Appendix 2 Complete list of all analyzed forms with title, number of concepts, and Unified Medical Language System coverage.

Multimedia Appendix 3 Frequencies of all analyzed concepts. List of medical concepts and subconcepts, suggested Unified Medical Language System codes, datatypes, and units of measurement, where applicable, sorted by frequency.

Multimedia Appendix 4 Generated revised list of common data elements for acute coronary syndrome.

## Abbreviations

ACCF: American College of Cardiology Foundation
ACS: acute coronary syndrome
Afreq: absolute frequency
AHA: American Heart Association
AHA/ACC: American Heart Association/American College of Cardiology
AHA/ACCF: American Heart Association/American College of Cardiology Foundation
ALKK: Arbeitsgemeinschaft Leitender Kardiologischer Krankenhausarzte
CABG: coronary artery bypass graft
CARDS: Cardiology Audit and Registration Data Standards
CCS: Canadian Cardiovascular Society
CDE: common data element
CDSS: clinical decision support systems
CPU: central processing unit
CRF: case report form
ECG: electrocardiogram
ESC: European Society for Cardiology
FHIR: Fast Healthcare Interoperability Resources
HL7: Health Level Seven
MI: myocardial infarction
NSTEMI: non-ST elevated myocardial infarction
ODM: Operational Data Model
PCI: percutaneous coronary intervention
QA: quality assurance
Rfreq: relative frequency
STEMI: ST elevated myocardial infarction
TIA: transient ischemic attack
UAP: unstable angina pectoris
UMLS: Unified Medical Language System
