# Supplementary material for: Common Data Elements for Acute Coronary Syndrome: Analysis Based on the Unified Medical Language System
Source: JMIR Med Inform. 2019 Aug 23;7(3):e14107. doi: 10.2196/14107 (PMC6729118; doi:10.2196/14107)
Supplement: Multimedia Appendix 1 [file medinform_v7i3e14107_app1.pdf]

SUPPLEMENT 1: LIST OF ALL CONTACTED REGISTRIES WITH RESPONSE

**Name**	**Weblink**	**Contact date (dd.mm.yyyy)**	**Result**
Acute Coronary Syndrome Quality Improvement in Kerala (ACS QUIK)	http://www.csikerala.org/acsregistry.php	19.03.2015	Included
Deutsches CPU-Register	https://cpu.dgk.org/cpu-register/	18.06.2015	Included
PCI Register der ALKK	https://stiftung-ihf.de/index.php/de/projekte/projekte-2014/pci-register	18.06.2015	Included
European Society of Cardiology (ACS snapshot survey, ESCARDIO ACS Survey II, ESCARDIO ACS Survey I, Acute coronary syndrome registry	https://www.escardio.org/Research/Registries-&-surveys/Observational-research-programme/registry-overview	10.03.2015	Refused
Scottish Heart Health Extended Cohort (SHHEC)	http://assign-score.com/about/shhec/	10.03.2015	No answer
PL-ACS	http://www.ncbi.nlm.nih.gov/pubmed/17853315	19.03.2015	No answer
NCVD-ACS	http://www.acrm.org.my/ncvd/registries_acs.php	19.03.2015	No answer
Jakarta Acute Coronary Syndrome Registry	https://clinicaltrials.gov/ct2/show/NCT02319473	19.03.2015	No answer
TRACS	http://www.thaiheart.org/images/sub_1291456609/TRACS.pdf	19.03.2015	No answer
Brazilian Registry of Acute Coronary Syndrome	http://www.scielo.br/pdf/abc/2013nahead/en_aop_4817.pdf	19.03.2015	No answer

French Registry of ACS (FAST-MI)	https://clinicaltrials.gov/ct2/show/NCT00673036	19.03.2015	No answer
PROCAM	https://www.ncbi.nlm.nih.gov/pubmed/3202078	19.03.2015	No answer
China Acute Myocardial Infarction (CAMI) Registry	https://www.ncbi.nlm.nih.gov/pubmed/27179740	18.06.2015	No answer
CRUSADE registry	http://www.emcreg.org/	10.03.2015	No answer
Herzinfarktregister Rheinland-Pfalz	https://msagd.rlp.de/de/unsere-themen/gesundheit-und-pflege/gesundheitliche-versorgung/herzinfarktregister/	18.06.2015	No answer
