# Supplementary material for: Common Data Elements for Acute Coronary Syndrome: Analysis Based on the Unified Medical Language System
Source: JMIR Med Inform. 2019 Aug 23;7(3):e14107. doi: 10.2196/14107 (PMC6729118; doi:10.2196/14107)
Supplement: Multimedia Appendix 2 [file medinform_v7i3e14107_app2.pdf]

1 TABLE A2: LIST OF ALL ANALYSED FORMS

Form name	Number of concepts	Annotated concepts	UMLS Coverage
ACCF/AHA ACS Key Data Elements	308	294	94%
ACCF/AHA ACS Key Data Elements - Primary cause of death	3	3	100%
CARDS	109	222	99%
Cardiology admission orders, physicians order sheet	69	75	100%
	489	594	98%
GRACE 2.0 ACS Risk Score	10	10	100%
ASSIGN Cardiovascular Risk Score Version 1.5	11	13	100%
Reynolds Risk Score	8	8	100%
QRISK2 CVD Score	14	14	100%
Framingham Risk Score	7	9	100%
TIMI Risk Score for STEMI	8	8	100%
TIMI Risk Score for UA/NSTEMI	7	7	100%
SCORE	5	5	100%
CRUSADE	8	8	100%
ACTION	8	11	100%
	86	93	100%
GRACE	193	213	97%
ACTION Registry	186	169	88%
STEMI-Register Münster	83	120	98%
Kerala ACS Registry	71	74	94%
Deutsches CPU-Register	157	190	96%
ALKK-PCI Register	146	110	86%
SWEDEHEART	229	202	100%
	1065	1078	94%

**Quality assurance Coronary Angiography and Percuteanous Intervention (PCI) - Basic (AQUA)**	32	29	97%
**Quality assurance Coronary Angiography and Percuteanous Intervention (PCI) -Procedure (AQUA)**	15	8	80%
**Quality assurance Coronary Angiography and Percuteanous Intervention (PCI) -PCI (AQUA)**	19	7	58%
**Quality assurance Coronary Angiography and Percuteanous Intervention (PCI) - Coronary Angiography (AQUA)**	5	4	80%
**Quality assurance Carotid Revascularization - Interventional/Catheter-assisted (PTA and/or Stent) (AQUA)**	10	5	70%
**AMI Specifications Manual for National Hospital Inpatient Quality Measures**	42	28	88%
	123	81	79%
**Münster - Labor**	22	22	100%
**Münster - Echokardiographie Anmeldung**	11	10	100%
**Münster - Echokardiographie Befund**	16	16	100%
**Münster - EKG Befund**	11	11	100%
**Münster - Arztbrief**	14	14	100%
**Münster - Angiographie Anmeldung**	44	51	98%
	118	124	100%
**Bremen - Admission form**	64	54	98%
**Bremen - Herzkatheterbericht**	26	25	96%
**Bremen - EKG**	14	13	93%
**Bremen - Labor**	19	19	100%
**Bremen - Transösophageale Echokardiographie**	16	16	100%
**Bremen - Transthorakale Echokardiographie**	15	15	100%
	154	142	98%
**Dresden - Arztbrief**	14	14	100%
**Dresden - Operationsprotokoll (Herzklappen)**	13	10	77%
**Dresden - Herzkatheterbericht**	14	13	93%
**Dresden - Echobericht**	15	15	100%
**Dresden - Labor**	35	35	100%

**Dresden - Admission form**	54	54	100%
	145	141	95%
**Magdeburg - Arztbrief**	11	11	100%
**Magdeburg - Labor**	29	29	100%
**Magdeburg - Anamnese**	63	61	100%
**Magdeburg - Echoanmeldung**	7	7	100%
**Magdeburg - EKG**	8	8	100%
**Magdeburg - Herzkatheterbericht**	16	15	94%
	134	131	99%
**PLATO**	514	489	93%
	514	489	93%
**ATLAS**	310	195	96%
	310	195	96%
**[Eligibility] EARLY ACS: Early Glycoprotein IIb/IIIa Inhibition in Patients With Non-ST-segment Elevation Acute Coronary Syndrome (Study P03684AM2)**	16	16	100%
**[Eligibility] PLATO**	7	7	100%
**[Eligibility] Effectiveness And Safety Of Dalteparin In Patients With Acute Coronary Syndromes Without ST Elevations In Clinical Practice**	8	8	100%
**[Eligibility] Study to Assess Safety and Efficacy of Ticagrelor (AZD6140) Versus Clopidogrel in Asian/Japanese Patients With Non-ST or ST Elevation Acute Coronary Syndromes (ACS)**	7	7	100%
**[Eligibility] ATLAS**	12	12	100%
**[Eligibility] An Efficacy and Safety Study for Rivaroxaban in Patients With Acute Coronary Syndrome**	6	6	100%
**[Eligibility] ALPACS**	4	4	100%
**[Eligibility] Efficacy and Safety of Aliskiren and Valsartan Versus Placebo in Patients Stabilized Following an Acute Coronary Syndrome**	9	9	100%
**[Eligibility] Clopidogrel to Prasugrel in Acute Coronary Syndrome (ACS) Patients**	29	29	100%
**[Eligibility] Early Use of Rosuvastatin in Acute Coronary Syndromes:**	20	20	100%

**Targeting Platelet-Leukocyte Interactions**			
**[Eligibility] A Comparison of Prasugrel and Clopidogrel in Acute Coronary Syndrome Subjects (TRILOGY ACS)**	16	16	100%
**[Eligibility] A Comparison of Antiplatelet Therapies in Asian Subjects With Acute Coronary Syndrome**	22	22	100%
**[Eligibility] Phase 2 Study in Vascular Inflammation on Patients After an Acute Coronary Syndrome Event**	23	23	100%
**[Eligibility] A Comparison of Prasugrel (CS-747) and Clopidogrel in Acute Coronary Syndrome Subjects Who Are to Undergo Percutaneous Coronary Intervention**	16	16	100%
**[Eligibility] A Phase IV Study of the Onset and Maintenance of the Antiplatelet Effect of Ticagrelor Compared With Clopidogrel in Chinese Patients With ACS (HouYi)**	16	16	100%
**[Eligibility] Patient-Centered Adherence Intervention After Acute Coronary Syndrome (ACS) Hospitalization (MEDICATION)**	6	6	100%
**[Eligibility] The Effect on Blood Cells, Known as Platelets, Using Prasugrel vs Clopidogrel in Patients With the Heart Problem Acute Coronary Syndrome (ACS)**	15	15	100%
**[Eligibility] Effect of Prasugrel on Platelets After One Week in Patients Already Taking Clopidogrel After a Cardiac Event (SWAP)**	18	17	94%
**[Eligibility] Switching From Fondaparinux to Bivalirudin or Unfractionated Heparin in ACS Patients Undergoing PCI (SWITCHIII)**	36	36	100%
**[Eligibility] Randomized Investigation of Chest Pain Diagnostic Strategies**	22	22	100%
**[Eligibility] Mojito Study (Mashed Or Just Integral Pill of TicagrelOr ?)**	29	29	100%
**[Eligibility] A Comparison of Prasugrel at PCI or Time of Diagnosis of Non-ST Elevation Myocardial Infarction (ACCOAST)**	15	14	93%
**[Eligibility] Early Versus Delayed Timing of Intervention in Patients With Acute Coronary Syndromes (TIMACS)**	10	9	100%
**[Eligibility] Fondaparinux Trial With Unfractionated Heparin (UFH) During Revascularization in Acute Coronary Syndromes (ACS) (FUTURA/OASIS 8)**	32	32	100%
**[Eligibility] Evaluation of a New Cardiac Biomarker Assay**	10	10	100%
**[Eligibility] Multicenter Study to Rule Out Myocardial Infarction by Cardiac Computed Tomography (ROMICAT-II)**	35	35	100%
**[Eligibility] Perioperative Management of Patients With Coronary Stents Undergoing Noncardiac Surgery (RegistStents)**	12	12	100%
**[Eligibility] A Clinical Trial Comparing Cangrelor to Clopidogrel Standard Therapy in Subjects Who Require Percutaneous Coronary Intervention**	24	24	100%

**(PCI) (CHAMPION PHOENIX)**			
**[Eligibility] Maintenance of Platelet Inhibition With Cangrelor (Bridge)**	9	9	100%
**[Eligibility] Trial to Assess the Safety and Effects of Vorapaxar in Japanese Subjects With Acute Coronary Syndrome**	28	28	100%
**[Eligibility] RE-DEEM Dose Finding Study for Dabigatran Etexilate in Patients With Acute Coronary Syndrome**	10	10	100%
**[Eligibility] Risk Markers in the Acute Coronary Syndromes (RACS)**	7	7	100%
**[Eligibility] Clinical Effects of Eptifibatide Administration in High Risk Patients Presenting With Non-ST Segment Elevation Acute Coronary Syndrome (NSTE-ACS) Requiring Urgent Coronary Artery Bypass Graft Surgery in Short- and Long-Term Follow-up**	26	26	100%
**[Eligibility] Myocardial Ischemia and Transfusion (MINT)**	17	17	100%
	572	569	100%
	3710	3637	97%
