# Supplementary material for: Common Data Elements for Acute Coronary Syndrome: Analysis Based on the Unified Medical Language System
Source: JMIR Med Inform. 2019 Aug 23;7(3):e14107. doi: 10.2196/14107 (PMC6729118; doi:10.2196/14107)
Supplement: Multimedia Appendix 3 [file medinform_v7i3e14107_app3.pdf]

1 TABLE A3: FREQUENCIES OF ALL ANALYZED CONCEPTS

Suggested UMLS-Code	Concept Name	Included Concepts	Data-Type	Codelist	Unit of measurement	Absolute Frequency
**C1532338**	Percutaneous Coronary Intervention (PCI)	History of PCI, Percutaneous coronary revascularization, History of revascularization, Reason for delay in PCI, Date of most recent PCI, Indication for PCI, Contraindication for PCI, Reperfusion therapy, Primary PCI, Patient refused PCI, Total number of PCI procedures, Date of Coronary Revascularization, Reason for Revascularization, Urgent revascularization				98
**C0038454**	Stroke/TIA	History of Stroke, TIA, History of TIA, Type of stroke, Date of most recent stroke, Hemorrhagic stroke	Bool			77
**C0001779**	Date of birth	Age	DateTime			73
**C0019080**	Hemorrhage other than stroke	Gastrointestinal bleeding, Major bleed, Minor bleed, Intracranial hemorrhage, History of bleeding from GI system, Bleeding treatment, Date of hemorrhage, Bleeding location, Spontaneous bleed, Re-operation due to bleeding	Bool			69
**C0010055**	Coronary Artery Bypass	History of CABG, Date of most				53

	Graft (CABG)	recent CABG, Date of CABG				
**C0002962**	Angina pectoris	Unstable angina pectoris, CCS Classification, History of Angina, Angina, Stable angina pectoris, Secondary cause of angina	Bool			48
**C0871470**	Blood pressure	Systolic Bloog Pressure, Diastolic Blood Pressure, Blood Pressure	ItemGroup			47
**C1306577**	Death	Date of death, Cause of death, Time of Death, Survival status at discharge, Discharge status, Death witnessed	ItemGroup			47
**C0085532**	Coronary Angiography	Cardiac Catheterization, Lävokardiographie, Prior catheterization, Indication for coronary angiography, Date of Coronary angiography, Contraindication for angiography				46
**C0027051**	Myocardial infarction	History of myocardial infarction, Date of most recent MI, Acute MI, Isolated posterior MI, Recurrent MI, Posterior Infarction	Bool			44
**C0018801**	Heart failure	CHF, History of heart failure, Date of heart failure, Heart failure, Dekomp. Herzinsuffizienz	Bool			43
**C0004057**	Aspirin/ASS	Aspirin contraindication, Asprin discharge, Aspirin in first 24h,				42

		Aspirin dose				
**C0040044**	Thrombolytics	Thrombolytics contraindication, Fibrinolytics, Date of Fibrinolytic, Thrombolytic dosage, Plättchenhemmer				42
**C0523952**	Troponin	Troponin I, Troponin T, Peak troponin value, Troponin T ULN, Troponin I ULN	Float			40
**C1299487**	Patient name	Patient initials, Patient middle name, Patient first name, Patient last name	Text			40
**C0070166**	Clopidogrel	Clopidogrel contraindicated, Clopidogrel dosage, Clopidogrel (discharge), Clopidogrel time, start time				36
**C0011849**	Diabetes mellitus	Diabetes therapy, Insulin use, Insulin, Oral diabetes treatment	Bool			31
**C0523584**	CK-MB	CK-MB ULN, CK-MB peak value	Float			31
**C0018810**	Heart rate		Float		bpm	30
**C0151744**	Myocardial ischemia	ECG signs of myocardial ischemia, ECG abnormal for ischemia, Recurrent ischemic symptoms, Number of all other episodes of cardiac ischaemic symptoms	Bool			30
**C0003280**	Anticoagulation	Oral anticoagulants, Contraindication to anticoagulation, Anticoagulant therapy, Indication for				29

		anticoagulation, Anticoagulant (parenteral), Anticoagulant start date, Stop date, Dose				
**C0013798**	Electrocardiogram	Time of ECG, ECG time, ECG findings				28
**C0021430**	Informed consent	Date signed informed consent	Bool			28
**C0520886**	ST elevation	Anterior ST elevation, Inferior ST elevation, Lateral ST elevation	Bool			28
**C0543467**	Surgical procedure (other than CABG)	History of surgery, Non-cardiovascular invasive procedure				28
**C0079399**	Gender		CodeList	Male, Female, Other		27
**C0005910**	Weight		Float		kg	25
**C0304516**	Beta-Blocker	Beta-Blocker contraindication				25
**C0549206**	Pregnancy	Pregnant woman, Pregnancy test	Bool			25
**C0948089**	Acute Coronary Syndrome		Bool			25
**C1384495**	Myocardial stress test	Non-invasive stress test, Stress echocardiography, Stress test pos/neg, Stress test date, Date of stress test				25
**C1536220**	STEMI	Anterior STEMI, STEMI date	Bool			25
**C0035078**	Impaired renal function	Renal insufficiency, Renal failure, Chronic renal disease, Renal insufficiency	Bool			24
**C3536766**	Low molecular weight	Enoxaparin, LMWH contraindication, LMWH start				24

	heparin	date, LMWH dose				
**C0036980**	Cardiogenic shock	IV Cardiogenic shock: Signs include hypotension (systolic pressure of 9 mmHg or less) and evidence of peripheral vasoconstriction such as oliguria, cyanosis and diaphoresis, Date of cardiogenic shock	Bool			23
**C0360714**	Statins	Statin contraindicated, Statins before admission, Statin dose				23
**C1620287**	Prasugrel	Prasugrel Dose, Prasugrel contraindicated, Prasugel start time				23
**C0003015**	ACE Inhibitors	ACE inhibitor contraindicated				22
**C3640054**	Glycoprotein 2b/3a inhibitors	IIb/IIIa blocker dosage, IIb/IIIa start time, Reason for GP .. Administration, Stop Date, Contraindiacted				22
**C0005890**	Height		Float		cm	21
**C0020538**	Hypertension	History of Hypertension, Hypertensive crisis	Bool			21
**C0232187**	Cardiac rhythm	Sinus rhythm, cardiac arrhythmia, Atrial arrhythmia	CodeList	Sinus rhythm, Atrial fibrillation or flutter, Paced, Other rhythm		21
**C0428772**	Left ventricular ejection fraction	LVEF planned for after discharge, Linksventrikuläre Funktion, LVEF assessed	Float		%	21
**C0520887**	ST depression	Inferior ST depression, Anterior ST depression, Lateral ST	Bool			21

		depression				
**C0521942**	Angiotensin 2 Receptor Antagonists	ARB Contraindication, ARC contraindicated				21
**C1999375**	Ticagrelor	Ticagrelor dosage, Ticagrelor contraindication, Ticagrelor start time				21
**C2825026**	Unfractionated heparin	Unfractionated heparin contraindication, Unfractionated heparin dose, Unfractionated heparin start time				21
**C0013516**	Echocardiography	Date of Echocardiogram				20
**C0030163**	Cardiac Pacemaker	Pacemaker implantation, Paced rhythm, Permanent pacemaker, Temporary pacemaker				25
**C0201976**	Creatinine	Peak Creatinine	Float			20
**C0543414**	Tobacco consume		Bool			20
**C2584899**	Date of examination					20
**C0019046**	Hemoglobin	Anaemia	Float			18
**C1320532**	Arrival time at hospital/ER	Arrival date, Presentation date/time	DateTime			18
**C3537184**	NSTEMI		Bool			18
**C2361123**	Date of discharge	Time of hospital discharge for index event	DateTime			17
**C1320528**	Time of symptom onset	Duration of symptoms, Onset date of index event	DateTime			16
**C1879316**	Transfusion of blood	Date of first transfusion, Declines blood transfusion				16

**C0021860**	Intra-aortic balloon pump					15
**C0043031**	Warfarin	Warfarin contraindicated				15
**C0429090**	Significant Q waves	Q waves in anterior leads, Q waves in inferior leads, significant Q waves in anterior leads	Bool			15
**C1273352**	Intracardial defibrillator (ICD)	Temporary pacemaker ICD, ICD implantation date				15
**C0023211**	Left bundle branch block		Bool			14
**C0085605**	Impaired hepatic function	Chonric liver disease	Bool			14
**C1302393**	Date of admission		DateTime			14
**C0011900**	Diagnosis	Präoperative diagnosis	Text			13
**C0013404**	Dyspnea		Bool			13
**C0018821**	Cardiac surgery					13
**C0040405**	X -ray tomography	CT location				0
**C0525032**	INR	Quick, Quick	Float			13
**C1553892**	Current medication list	On blood pressure treatment, Medication list				13
**C3251812**	Increased bleeding risk		Bool			13
**C0004238**	Atrial fibrillation	Atrial fibrillation date, Vfib date	Bool			12
**C0008031**	Chest pain		Bool			12
**C0018790**	Cardiac arrest	Date of cardiac arrest, Cardiac	Bool			12

		arrest outsite facility				
**C0019993**	Hospitalization since previous visit		Bool			12
**C0024485**	MRI imaging	Contraindications to MRI				6
**C0455404**	Family history of coronary heart disease	Familienanamnese	Bool			12
**C0017887**	Nitrates	Oral Nitrates, Amount of sublingual nitrates, Topical nitrates, Nitroglycerin	Float			11
**C0020517**	Allergies	Allergy to study drug	Text			11
**C0087153**	Ventilator					11
**C0190658**	Pulmonary Artery Catheter	Swan Gans Catheter				11
**C0201973**	Creatine Kinase	CK maximum value, CK ULN	Float			11
**C0421449**	Patient address	Street	Text			11
**C0520888**	Pathological T wave	T inversion	Bool			11
**C0013801**	Holter monitoring					10
**C0168273**	Bivalirudin	Bivalirudin contraindicated, Bivalirudin start time				10
**C0220825**	Evaluation					10
**C0242339**	Dyslipidaemia	Hyperlipidemia	Float			10
**C0428472**	LDL Cholesterol		Float			10
**C0679831**	Patient history	Medical history				10
**C1445957**	Total cholesterol	Hypercholesterinemia	Float			10

**C1546432**	Transfer from other hospital	Date of transfer, Time of transfer	DateTime			10
**C1550330**	Urgency	Urgent procedure				10
**C1881056**	Peripheral arterial disease		Bool			10
**C3272316**	Cardiac stent placement	Insertion of Drug Coated Stent(s), Stent type				10
**C0005821**	Platelet count	Thrombocytopenia	Float			9
**C0006826**	Malignom	Intracranial neoplasm, Tumorerkrankungen	Bool			9
**C0085612**	Ventricular arrhythmia		Bool			9
**C0151942**	Other arterial thrombotic event		Bool			9
**C0181598**	Left ventricular assist device		Bool			9
**C0242231**	Coronary stenosis	RCA, PDA, RPL and AM branches percent stenosis	Bool			9
**C0332144**	Final/discharge diagnosis		Text			9
**C0421454**	Post code		Text			9
**C0600290**	Rehospitalization	Readmission reason, Readmission date, Gesamtzahl aller Rehospitalisationen, Date of Readmission	ItemGroup			9
**C0677490**	Pulmonary angiography					9
**C0877248**	Adverse events		Text			9

**C1096021**	Antiplatelet therapy					9
**C1320303**	Visit date		DateTime			9
**C1446476**	Electrophysiology study		Bool			9
**C2348585**	Patient ID		Text			9
**C3177142**	Symptoms at admission	Reason for hospitalization, Primary reason for hospitalization, Clinical symptoms	Text			9
**C0002007**	Aldosterone Blocking Agent	Aldosterone blocking agent contraindicated, Dose				8
**C0012798**	Diuretics					8
**C0040207**	Ticlopidine	Ticlopidine dose, Ticlopidin contraindicated				8
**C0201657**	CRP		Float			8
**C0560738**	Ventilation/perfusion scan					8
**C0581603**	Revascularization					8
**C0589120**	Follow up	Follow up date	DateTime			8
**C0948369**	Reinfarction	Reinfarction date	Bool			8
**C2348568**	Study participation status		Text			8
**C2361125**	Attending physician name	Physician name, Physician name, Resident physician name	Text			8
**C3272266**	TIMI Score		Integer			8
**C0003367**	Non-statin lipid					7

	lowering agent					
**C0007203**	Cardiopulmonary Resuscitation					7
**C0019004**	Hemodialysis					7
**C0232326**	ST-segment changes	Nonspecific ST/T Change, ST deviation	Bool			7
**C0332135**	Working diagnosis on admission	Führende Diagnosie nach Herzkatheter	Text			7
**C0428474**	Serum LDL measurement		Float			7
**C0700589**	Contraception	Kontrazeptiva				7
**C0948268**	Hemodynamic instability	Hemodynamics	Bool			7
**C1098510**	Fondaparinux	Contraindication to fondaparinux				7
**C1271630**	Cardiac biomarker measurements		Float			7
**C1299364**	Culprit artery	Bypass stenosis	CodeList	LAD, LCx, RCA, LM, Graft/Stent,Unknown		7
**C1956346**	Coronary Artery Disease	History of Coronary Artery disease	Bool			7
**C0001948**	Alcohol consumption	History of alcohol consumption	Text			6
**C0002940**	Aneurysm	Aneurysma, Intracranial aneurysm	Bool			6
**C0015031**	Ethnic group		CodeList			6
**C0018935**	Hematocrit		Float			6

**C0019018**	HbA1c		Float			6
**C0021672**	Insurance		Text			6
**C0030605**	PTT		Float			6
**C0034510**	Race		CodeList			6
**C0202042**	Blood Glucose	Fasting Glucose	Float			6
**C0202236**	Triglycerides		Float			6
**C2024081**	Number of stents	Number of drug eluting stents, Number of bare metal stents				6
**C2024776**	Cardiac risk factors	History and risk factors, Cardiovascular risk factors, risk factor for cardiovascular complication	Bool			6
**C2348066**	Dabigatran	Dabigatran contraindicated				6
**C2983649**	QRS annotation	QRS duration	Bool			6
**C3258270**	Means of transport	Means of transfer				6
**C0003211**	NSAIDs (other than Aspirin)					5
**C0003857**	Arteriovenous malformation		Bool			5
**C0017654**	GFR		Float			5
**C0023508**	White blood cell count	Leukozyten	Float			5
**C0031809**	Physical examination					5
**C0040034**	Thrombocytopenia		Float			5
**C0087111**	Procedure					5

**C0243095**	Findings					5
**C0455829**	Waist circumference		Float		cm	5
**C1515258**	Telephone number		Text			5
**C1858274**	Reduced life expectancy		Bool			5
**C2315323**	Indication					5
**C2936588**	Thienopyridine					5
**C3275120**	Number of diseased vessels		Integer			5
**C3838354**	Arrival time at Cath Lab		DateTime			5
**C0004245**	AV block		Bool			4
**C0006684**	Calcium channel blocker					4
**C0007177**	Cardiac Tamponade		Bool			4
**C0013604**	Edema		Bool			4
**C0014772**	Red blood cell count		Float			4
**C0015259**	Exercise	Excercise counceling				4
**C0018818**	Ventricular Septal Defect	Septal defect	Bool			4
**C0021641**	Insulin					4
**C0042514**	Ventricular tachycardia	Vtach date	Bool			4
**C0201836**	Alanine aminotransferase		Float			4
**C0201899**	Aspartate aminotransferase		Float			4

**C0202230**	TSH		Float			4
**C0253563**	Eptifibatide					4
**C0337443**	Sodium		Float			4
**C0369183**	MCH		Float			4
**C0421456**	Occupation	Berufsanamnese	Text			4
**C0474535**	MCHC		Float			4
**C0570562**	Allergy to contrast medium		Bool			4
**C0700431**	Cardiac rehabilitation					4
**C0741921**	Cardiac markers elevated		Float			4
**C1273715**	Smoking counseling					4
**C1948043**	MCV		Float			4
**C2267235**	Vitamin-K antagonists					4
**C2348570**	Premature termination of study		Bool			4
**C3272298**	Arterial access site					4
**C3536847**	Oral Thrombin inhibitor					4
**[C0023303, C0021708]**	Days spent in ICU		Integer			3
**[C0184666, C0450429]**	Admission location					3
**[C3261085, C1264639]**	EMS dispatch time		DateTime			3

**C0004096**	Asthma		Bool			3
**C0004239**	Atrial Flutter		Bool			3
**C0005902**	Body surface area		Float		qm	3
**C0009566**	Complications		Text			3
**C0009924**	Contrast medium	Kontrastmittelmenge				3
**C0011946**	Dialysis					3
**C0020443**	Hypercholesterolemia		Float			3
**C0024117**	COPD		Bool			3
**C0026266**	Mitral regurgitation		Bool			3
**C0031050**	Pericard					3
**C0031444**	Marcumar					3
**C0073633**	Ranolazine					3
**C0202194**	Potassium		Float			3
**C0206461**	Dalteparin	Dalteparin contraindicated				3
**C0304509**	Ionotropic agent					3
**C0332677**	Trauma	History of trauma	Bool			3
**C0360703**	Antiarrhythmics	Antiarhythmics (discharge)				3
**C0429028**	QT interval	QT	Text			3
**C0455713**	Type of procedure					3
**C0457617**	Mechanical support					3
**C0577789**	Aortic valve					3
**C0577791**	Mitral valve					3

**C0577797**	Tricupid valve					3
**C0745041**	Event prolonged hospitalization					3
**C0849535**	Lab results					3
**C1095989**	BNP		Float			3
**C1112720**	Unspecific ST changes		Bool			3
**C1305855**	Body mass index		Float		kg/qm	3
**C1510472**	Drug dependence	Documented or self-reported cocaine use within the past 48 hours (acute)	Bool			3
**C1739768**	Rivaroxaban	Contraindication				3
**C1831808**	Apixaban	Contraindication				3
**C1959586**	Cardiac Wall rupture	Cardiac rupture	Bool			3
**C2348485**	X-ray Exposure time					3
**C2733251**	EQ-5D-3L Quality of Life		Text			3
**[C0011008, C3495034]**	Time of transfer from Emergency department		DateTime			2
**C0003873**	Rheumatoid arthritis		Bool			2
**C0004398**	Autopsy performed		Bool			2
**C0004936**	Mental illness		Bool			2
**C0005903**	Body temperature		Float		°C	2
**C0007282**	Carotid stenosis		Bool			2
**C0007412**	Catecholamines					2

**C0010520**	Cyanosis		Bool			2
**C0012265**	Digoxin					2
**C0017319**	Primary physician		Text			2
**C0018823**	History of heart transplant					2
**C0020649**	Hypotension		Bool			2
**C0022671**	History of kidney transplant		Bool			2
**C0023303**	Lenght of stay		Integer			2
**C0030657**	Infectivity					2
**C0034065**	Pulmonary embolism		Bool			2
**C0035234**	Breathing sound		Text			2
**C0038418**	Streptokinase					2
**C0039070**	Syncope		Bool			2
**C0040338**	Snusning		Bool			2
**C0042071**	Urokinase					2
**C0042510**	Ventricular fibrillation		Bool			2
**C0085615**	Right branch bundle block		Bool			2
**C0150521**	Comfort measures only					2
**C0151814**	Coronary Occlusion		Bool			2
**C0161817**	Vascular complication		Text			2
**C0190173**	Valve replacement					2

**C0190211**	Angioplasty					2
**C0201913**	Total bilirubin		Float			2
**C0202035**	Gamma-glutamyl transpeptidase		Float			2
**C0235029**	Neurological complications		Text			2
**C0247025**	Tirofiban					2
**C0257190**	Ivabradin					2
**C0288672**	Abciximab					2
**C0332138**	Secondary diagnosis		Text			2
**C0340643**	Aortic dissection		Bool			2
**C0358591**	Proton pump inhibitors					2
**C0373595**	Creatinine Clearance		Float			2
**C0373675**	Magnesium		Float			2
**C0392209**	Nutritional status		Text			2
**C0428977**	Increased risk of bradycardia		Bool			2
**C0449462**	Stent length					2
**C0449494**	Type of graft					2
**C0450990**	Physical status		Text			2
**C0455503**	Depression		Bool			2
**C0489625**	QTc time		Float		ms	2
**C0523961**	Urea		Float			2

**C0553514**	Admission source		CodeList			2
**C0577786**	Left ventricle					2
**C0577787**	Right ventricle					2
**C0681902**	Laboratory results					2
**C0805839**	Date of last contact		DateTime			2
**C1148438**	Current condition					2
**C1167690**	PQ time		Float		ms	2
**C1269041**	Abdominal wall					2
**C1269890**	Right atrium					2
**C1269894**	Left atrium					2
**C1277187**	Left ventricular systolic dysfunction		Bool			2
**C1287726**	Puls status		Text			2
**C1291006**	Lung percussion					2
**C1291017**	Cardiac auscultation					2
**C1320531**	Time of first medical contact		DateTime			2
**C1322815**	Drug eluting balloon					2
**C1550373**	OPS-Codes		Text			2
**C2145202**	Brachytherapy					2
**C2700391**	Procedures					2
**C2825221**	Left main stenosis		Bool			2
**C2825222**	LAD Stenosis	Stenosis in LAD	Bool			2

**C3267166**	Right coronary artery					2
**C3532907**	Aorta					2
