# Supplementary material for: Common Data Elements for Acute Coronary Syndrome: Analysis Based on the Unified Medical Language System
Source: JMIR Med Inform. 2019 Aug 23;7(3):e14107. doi: 10.2196/14107 (PMC6729118; doi:10.2196/14107)
Supplement: Multimedia Appendix 4 [file medinform_v7i3e14107_app4.pdf]

1 TABLE A1: REVISED CDE LIST

Concepts Name	Suggested UMLS-Code	Data-Type	Codelist
**ACS-Timepoints**			
**Timepoints**			
**Date of admission**	C1302393	datetime	
**Date of discharge**	C2361123	datetime	
**Arrival at hospital**	C1320532	datetime	
**Time of symptom onset**	C1320528	datetime	
**Follow up date**	C3694716	datetime	
**Arrival time at Cath Lab**	C3838354	datetime	
**Days spent in ICU**	C0023303 C0021708	integer	
**EMS dispatch time**	C3261085 C1264639	datetime	
**Visit date**	C1320303	datetime	
**Lenght of stay**	C0023303	integer	
**Date of last contact**	C0805839	datetime	
**Time of first medical contact**	C1320531	datetime	
**Time of transfer from Emergency department**	C0011008 C3495034	datetime	
**Date of transfer**	C1546432	datetime	
**Means of transfer**	C0449375	text	
**Admission location**	C0450429	text	
**Patient data**			
**Patient data**			
**Date of birth**	C0001779	text	
**Gender**	C0079399	text	1=Male 2=Female 3=Other
**Informed consent**	C0021430	boolean	

**Tobacco consume**	C0543414	text	
**Family history of coronary heart disease**	C0455404	boolean	
**Patient address**	C0421449	text	
**Allergies**	C0020517	text	
**Patient ID**	C2348585	text	
**First name**	C1443235	text	
**Study Participation status**	C2348568	boolean	
**Insurance**	C0021672	text	
**Postal code**	C0421454	text	
**Ethnic Group**	C0015031	text	
**Race**	C0034510	text	
**Physician name**	C2361125	text	
**Telephone number**	C1515258	text	
**Reduced life expectancy**	C1858274	boolean	
**Allergy to contrast medium**	C0570562	boolean	
**Occupation**	C0028811	text	
**Hospitalization since previous visit**	C0019993	boolean	
**Premature termination of study**	C2348570	boolean	
**Primary physician**	C0017319	text	
**Admission source**	C0553514	text	
**OPS-Codes**	C1550373	text	
**Patient name**	C1299487		
**First name**		text	
**Last Name**		text	
**Initials**		text	
**Patient history**			
**Patient history**	C0679831		

**Alcohol consumption**	C0001948	boolean	
**History of kidney transplant**	C0022671	boolean	
**Increased risk of bradycardia**	C0428977	boolean	
**Dyslipidemia**	C0242339	text	
**Thrombocytopenia**		text	
**Hypercholesterolemia**		text	
**History of heart transplant**	C0018823	text	
**Exercise**	C0015259	boolean	
**Laboratory**			
**Troponin**	C0523952		
**Troponin T**	C1141947	float	ng/mL
**Troponin I**	C0920210	float	ng/mL
**Laboratory**	C0681902		
**Creatinine**	C0201976	float	mg/dL
**Hemoglobin**	C0019046	float	mmol/L
**CK-MB**	C0523584	float	ng/mL
**Total cholesterol**	C1445957	float	mmol/L
**INR**	C0525032	float	
**Creatine Kinase**	C0201973	float	U/L
**LDL Cholesterol**	C0202117	float	mmol/L
**Platelet count**	C0005821	float	/L
**CRP**	C0201657	float	mg/L
**LDL measurement**	C0428474	float	mmol/L
**PTT**	C0030605	float	s
**Triglycerides**	C0202236	float	mg/dL
**Hematocrit**	C0518014	float	%
**GFR**	C0017654	float	mL/min
**White blood cell count**	C0023508	float	/L
**Cardiac biomarker**	C1271630	text	

**measurements**			
**HbA1c**	C0019018	float	%
**Blood Glucose**	C0202042	float	mg/dL
**Alanine aminotransferase**	C0201836	float	U/L
**MCH**	C0369183	float	pg
**Cardiac markers elevated**	C0741921	boolean	
**Red blood cell count**	C0014772	float	/L
**Aspartate aminotransferase**	C0201899	float	IU/L
**TSH**	C0202230	float	mIU/L
**Sodium**	C0337443	float	mmol/L
**MCV**	C1948043	float	fL
**MCHC**	C0474535	float	g/dL
**BNP**	C1095989	float	pg/mL
**Potassium**	C0202194	float	mmol/L
**Total bilirubin**	C0201913	float	µmol/L
**Magnesium**	C0024467	float	mmol/L
**Creatinine Clearance**	C0373595	float	mL/min
**Gamma-glutamyl transpeptidase**	C1883012	float	U/L
**Urea**	C0523961	float	mmol/L
**Medication**			
**Medication**			
**Current medication list**	C1553892	text	
**Contraception**	C0700589	text	
**Diuretics**	C0012798	text	
**Thienopyridine**	C2936588	text	
**NSAIDs**	C0003211	text	
**Non-statin lipid lowering agent**	C0003367	text	
**Eptifibatide**	C0253563	text	
**Calcium channel blocker**	C0006684	text	

**Insulin**	C0021641	text	
**Oral Thrombin inhibitor**	C3536847	text	
**Vitamin-K antagonists**	C2267235	text	
**Ionotropic agent**	C0304509	text	
**Antiarrhythmics**	C0003195	text	
**Marcumar**	C0031444	text	
**Catecholamines**	C0007412	text	
**Tirofiban**	C0247025	text	
**Abciximab**	C0288672	text	
**Urokinase**	C0042071	text	
**Digoxin**	C0012265	text	
**Streptokinase**	C0038418	text	
**Ranolazine**	C0073633	text	
**Proton pump inhibitors**	C0358591	text	
**Ivabradin**	C0257190	text	
**Nitrates**	C0017887	text	
**Aspirin**	C0004057		
**Aspirin contraindication**	C0729798	boolean	
**Aspirin dosage**	C0178602 C0004057	float	mg
**Aspirin at discharge**	C3871203 C0004057	boolean	
**Anticoagulation**	C0003280		
**Anticoagulation start date**	C0003280 C0808070	datetime	
**Anticoagulation dose**	C0178602 C0003280	float	mg
**Anticoagulant**	C0003280	text	IV unfractionated=IV unfractionated Low molecular weight=Low molecular weight Bivalirudin=Bivalirudin Fondaparinux=Fondaparinux Other=Other
**Oral anticoagulant**	C0354604	boolean	
**Anticoagulation contraindicated**	C1531588	boolean	
**Thrombolytics**	C0040044		

**Thrombolytics contraindicated**	C3260558	boolean	
**Thrombolytics dosage**	C0040044 C0178602	float	
**Clopidogrel**	C0070166		
**Clopidogrel contraindicated**	C1319916	boolean	
**Clopidogrel dosage**	C0070166 C0178602	float	
**Clopidogrel start time**	C130188 C0070166	datetime	
**Low molecular weight heparin**	C0019139		
**Enoxaparin**	C0206460	text	
**LMWH start date**	C3536766 C1301880	datetime	
**LMWH dose**	C3536766 C0178602	float	
**Glycoprotein 2b/3a inhibitors**	C3640054		
**2b/3a blocker dosage**	C3640054 C0178602	float	
**2b/3a start time**	C1301880 C3640054	datetime	
**Unfractionated heparin**	C2825026		
**Unfractionated heparin contraindication**	C2825026 C0522473	boolean	
**Unfractionated heparin dose**	C2825026 C0178602	float	
**Unfractionated heparin start time**	C1301880 C2825026	datetime	
**Beta-Blocker**	C0001645		
**Beta-Blocker contraindication**	C1278474	boolean	
**Statins**	C0360714		
**Statin contraindicated**	C1277178	boolean	
**Statin dose**	C0360714 C0178602	float	
**ACE Inhibitors**	C0003015		
**ACE inhibitor contraindicated**	C0003015 C0522473	boolean	

**Angiotensin 2 Receptor Antagonists**	C0521942		
**ARB Contraindication**	C1273465	boolean	
**Warfarin**	C0043031		
**Warfarin contraindicated**	C0043031 C0522473	boolean	
**Prasugrel**	C1620287		
**Prasugrel Dose**	C1620287 C0178602	float	
**Prasugrel contraindicated**	C1620287 C0522473	boolean	
**Prasugel start time**	C1620287 C1301880	datetime	
**Ticagrelor**	C1999375		
**Ticagrelor dosage**	C1999375 C0178602	float	
**Ticagrelor contraindication**	C1999375 C0522473	boolean	
**Ticagrelor start time**	C1999375 C1301880	datetime	
**Bivalirudin**	C0168273		
**Bivalirudin contraindicated**	C0168273 C0522473	boolean	
**Bivalirudin start time**	C0168273 C1301880	datetime	
**Fondaparinux**	C1098510		
**Contraindication to fondaparinux**	C0700589 C0522473	boolean	
**Ticlopidine**	C0040207		
**Ticlopidine dose**	C0040207 C0178602	float	
**Ticlopidin contraindicated**	C0522473 C0040207	boolean	
**Dabigatran**	C2348066		
**Dabigatran contraindicated**	C2348066 C0522473	boolean	

**Aldosterone Blocking Agent**	C0002007		
**Aldosterone blocking agent contraindicated**	C0002007 C0522473	boolean	
**Aldosterone blocking agent dose**	C0002007 C0178602	float	
**Rivaroxaban**	C1739768		
**Rivaroxaban contraindicated**	C1739768 C0522473	boolean	
**Dalteparin**	C0206461		
**Dalteparin contraindicated**	C0206461 C0522473	boolean	
**Apixaban**	C1831808		
**Apixabon contraindicated**	C1831808 C0522473	boolean	
**Outcome**			
**Outcome**			
**Adverse events**	C0877248	text	
**Event prolonged hospitalization**	C0745041	text	
**Complications**	C0009566		
**Vascular complications**	C1393529	text	
**Neurological complications**	C0235029	text	
**Death**	C0011065		
**Discharge Status**	C0586514	text	1=Death 2=Alive 3=Unknown
**Cause of Death**	C0007465	text	
**Date of death**	C1148348	datetime	
**Autopsy performed**	C0004398	boolean	
**Rehospitalization**	C0600290		

**Reason for Rehospitalization**	C1830395	text	
**Date of Rehospitalization**	C1264639 C0600290	datetime	
**Rehabilitation**			
**Cardiac rehabilitation**	C0700431	boolean	
**Smoking counseling**	C1273715	boolean	
**Procedures**			
**Procedures**	C0087111		
**Intra-aortic balloon pump**	C0021860	boolean	
**Cardiac placemaker**	C0030163	boolean	
**Hemodialysis**	C0019004	boolean	
**Cardiac surgery**	C0018821	text	
**Pulmonary Artery Catheter**	C0190658	text	
**Ventilator**	C0087153	text	
**Arterial Access Site**	C3272298	text	1=Femoral 2=Brachial 3=Radial 4=Other
**Cardiopulmonary Resuscitation**	C0007203	boolean	
**Contrast medium**	C0009924	text	
**X-ray tomography**	C0040405	text	
**X-ray Exposure time**	C2348485	time	
**Mechanical support**	C0457617	text	
**MRI imaging**	C0024485	text	
**Holter monitoring**	C0013801	text	
**Pulmonary angiography**	C0677490	text	
**Hemodialysis**	C0019004	text	
**Drug eluting balloon**	C1322815	text	
**Valve replacement**	C0190173	text	
**Brachytherapy**	C0006098	text	
**Comfort measures only**	C0150521	boolean	
**Angioplasty**	C0162577	text	

**Revascularization**	C0581603	text	
**Ventilation/perfusion scan**	C0560738	text	
**Urgency**	C2188402	text	
**Percutaneous Coronary Intervention**	C1532338		
**History of PCI**	C1320647	boolean	
**Contraindication for PCI**	C0522473 C1532338	boolean	
**Date of most recent PCI**	C1532338 C1264639	datetime	
**Coronary Artery Bypass Graft**	C0010055		
**Date of CABG**	C2164190	datetime	
**History of CABG**	C1842251	boolean	
**Coronary Angiography**	C0085532		
**Date of Coronary angiography**	C0011008 C0085532	datetime	
**Contraindication for angiography**	C0085532 C0522473	boolean	
**Electrocardiogram**	C0013798		
**ECG findings**	C0438154	text	
**Time of ECG**	C2826759	datetime	
**Surgical procedure**	C0543467		
**History of surgery**	C0744961	boolean	
**Non-cardiovascular invasive procedure**	C1548804	text	
**Myocardial stress test**	C1384495		
**Date of stress test**	C1384495 C0011008	datetime	
**Stress echocardiography**	C0920208	text	
**Non-invasive stress test**	C3272313	text	

**Echocardiography**	C0013516		
**Date of Echocardiogram**	C0013516 C0011008	datetime	
**Cardiac stent placement**	C3272316		
**Stent type**	C0687568 C0332307	text	1=Drug-eluting stent[C1322815] 2=Bare metal stent[C2825200] 3=Other stent
**Number of stents**	C2024081	integer	
**Stent length**	C0449462	text	
**Transfusion of blood**	C0005841		
**Date of blood transfusion**	C1879316 C0011008	datetime	
**Intracardial defibrillator (ICD)**	C1273352		
**Temporary pacemaker ICD**	C0162589	boolean	
**ICD implantation date**	C1273352 C0011008	date	
**Examinations**			
**Examination findings**	C0243095		
**Pericard**	C0031050	text	
**Mitral valve**	C0577791	text	
**Tricupid valve**	C0577797	text	
**Aortic valve**	C0577789	text	
**Right atrium**	C1269890	text	
**Left atrium**	C1269894	text	
**Aorta**	C3532907	text	
**Cardiac auscultation**	C1291017	text	
**Right coronary artery**	C0226042	text	
**Right ventricle**	C0577787	text	
**Left ventricle**	C0577786	text	
**Left coronary artery**	C0226029	text	
**Physical examination**	C0031809	text	

**Abdominal wall**	C1269041	text	
**Lung percussion**	C1291006	text	
**Current condition**	C1148438	text	
**Weight**	C0005910	float	kg
**Heart rate**	C0018810	integer	bpm
**Pregnancy**	C0549206	boolean	
**Height**	C0005890	integer	cm
**Hypertension**	C0020538	boolean	
**Left ventricular ejection fraction**	C0428772	float	%
**Chest pain**	C0008031	boolean	
**Dyspnea**	C0013404	boolean	
**Peripheral arterial disease**	C1704436	boolean	
**Malignant neoplasm**	C0006826	boolean	
**Symptoms at admission**	C3177142	text	
**TIMI Score**	C3272266	text	
**Hemodynamic instability**	C0948268	boolean	
**Coronary Artery Disease**	C1956346	boolean	
**Cardiovascular risk factors**	C0850624	text	
**Aneurysm**	C0002940	text	
**Arteriovenous malformation**	C0003857	text	
**Waist circumference**	C0455829	integer	cm
**Ventricular Septal Defect**	C0018818	boolean	
**Edema**	C0013604	boolean	
**Cardiac Tamponade**	C0007177	boolean	
**Trauma**	C1368081	text	
**Body mass index**		float	kg/qm
**Body surface area**	C0005902	float	qm
**Cardiac Wall rupture**	C0018813	boolean	
**Mitral regurgitation**	C0026266	boolean	
**Drug dependence**	C1510472	text	
**Left ventricular assist device**	C0181598	boolean	
**COPD**	C0024117	boolean	

**Asthma**	C0004096	boolean	
**Pulmonary embolism**	C0034065	boolean	
**Carotid stenosis**	C0007282	boolean	
**Body temperature**	C0005903	float	°C
**Breathing sound**	C0035234	text	
**Aortic dissection**	C0340643	boolean	
**Rheumatoid arthritis**	C0003873	boolean	
**Cyanosis**	C0010520	boolean	
**Left ventricular systolic dysfunction**	C1277187	boolean	
**Syncope**	C0039070	boolean	
**Puls status**	C1287726	text	
**Nutritional status**	C0392209	text	
**Mental illness**	C0004936	boolean	
**Hypotension**	C0020649	boolean	
**Other arterial thrombotic event**	C0151942	boolean	
**EQ-5D-3L Quality of Life**	C2733251	text	
**Snusning**	C0040338	boolean	
**Infectivity**	C0030657	text	
**Date of examination**	C2826643	date	
**Electrophysiology study**	C1446476	text	
**Physical status**	C0450990	text	
**Blood pressure**	C0005823		
**Systolic blood pressure**	C0871470	integer	mmHg
**Diastolic blood pressure**	C0428883	integer	mmHg
**Myocardial ischemia**	C0151744		
**ECG signs of myocardial ischemia**	C1141959	boolean	
**Reinfarction**	C0948369		

**Reinfarction date**	C0948369 C0011008	datetime	
**Coronary stenosis**	C0242231		
**Stenosis percent**	C3897965	float	%
**Culprit artery**	C1299364	text	LAD=LAD LCx=LCx RCA=RCA LM=LM Graft/Stent=Graft/Stent Unknown=Unknown
**Number of diseased vessels**	C3275120	integer	
**Left main stenosis**	C2825221	boolean	
**LAD Stenosis**	C2825222	boolean	
**Diagnosis**			
**Stroke/TIA**	C0038454		
**History of stroke**	C0559159	boolean	
**Type of stroke**	C3897486	text	1=Ischemic 2=Hemorrhagic
**Date of stroke**	C0038454 C0011008	datetime	
**Heart failure**	C0018801		
**Heart failure**	C0018801		
**History of heart failure**	C0455531	boolean	
**Date of heart failure**	C0018801 C1264639	datetime	
**NYHA classification**	C1275491	text	1=I 2=II 3=III 4=IV
**Diagnosis**	C0011900		
**Acute Coronary Syndrome**	C0948089	boolean	
**Impaired renal function**	C0151746	boolean	
**STEMI**	C1536220	boolean	
**Cardiogenic shock**	C0036980	boolean	
**NSTEMI**	C3537184	boolean	
**Impaired hepatic function**	C0086565	boolean	
**Increased bleeding risk**	C3251812	boolean	

**Diagnosis**	C0011900	text	
**Atrial fibrillation**	C0004238	text	
**Working diagnosis on admission**	C0332135	text	
**Cardiac arrest**	C0018790	boolean	
**Final/discharge diagnosis**	C0332144	text	
**Secondary diagnosis**	C0332138	text	
**Depression**	C0011581	boolean	
**Hemorrhage**	C0019080		
**Bleeding location**	C0019080 C0450429	text	GI=GI Retroperitoneal=Retroperitoneal Intracranial=Intracranial Access Site=Access Site Other=Other GU=GU
**History of bleeding**	C3251812	boolean	
**Angina pectoris**	C0002962		
**CCS classification**	C1879987	text	I=I II=II III=III IV=IV
**History of Angina pectoris**	C0455530	boolean	
**Type of Angina pectoris**		text	1=Stable[C0340288] 2=Unstable[C0002965]
**Diabetes mellitus**	C0011849		
**Diabetes therapy**	C3274787	text	Insulin=Insulin Oral=Oral Diet=Diet None=None Other=Other
**Myocardial infarction**	C0027051		
**Infarction location**	C0027051 C0450429	text	Anterior=Anterior Posterior=Posterior Inferior=Inferior Other=Other
**History of MI**	C1275835	boolean	
**Date of most recent MI**	C2924287	datetime	
**ECG**			
**ECG findings**	C0438154		
**Cardiac rhythm**	C0232187	text	Sinus rhythm=Sinus rhythm[C0232201] Atrial fibrillation or flutter=Atrial fibrillation or flutter[C0004238 C0004239] Paced=Paced[C2981711] Other rhythm=Other rhythm

**Left bundle branch block**	C0023211	boolean	
**Ventricular arrhythmia**	C0085612	boolean	
**Significant Q waves**	C0429090	boolean	
**AV block**	C0004245	boolean	
**QT interval**	C0429028	text	
**Ventricular tachycardia**	C0042514	boolean	
**Ventricular fibrillation**	C0042510	boolean	
**PQ time**	C0429024	integer	ms
**Right branch bundle block**	C0085615	boolean	
**QTc time**	C0489625	integer	ms
**ST elevation**	C0520886		
**Location of ST elevation**		text	Anterior leads=Anterior leads[C3874460] Lateral leads=Lateral leads[C3874459] Inferior leads=Inferior leads[C3874457]
**ST depression**	C0520887		
**Location of ST depression**		text	Inferior leads=Inferior leads[C3874480] Anterior leads=Anterior leads[C3874467] Lateral leads=Lateral leads[C0743440]
**Pathological T wave**	C0438167		
**T inversion**	C0520888	boolean	
**ST-segment changes**	C0232326		
**ST deviation**	C1116089	boolean	
**Nonspecific ST/T Change**	C0920117	boolean	
**QRS annotation**	C0429098		
**QRS duration**	C0429025	integer	ms
